# Pharmacokinetics of the phosphatidylserine tracers ^99m^Tc-lactadherin and ^99m^Tc-annexin V in pigs

**DOI:** 10.1186/2191-219X-3-15

**Published:** 2013-03-09

**Authors:** Runa H Poulsen, Jan T Rasmussen, June Anita Ejlersen, Christian Flø, Lise Falborg, Christian W Heegaard, Michael Rehling

**Affiliations:** 1 Department for Clinical Medicine, Aarhus University Hospital, Skejby, Aarhus N 8200, Denmark; 2 Protein Chemistry Laboratory, Department of Molecular Biology and Genetics, Aarhus University, Aarhus 8000, Denmark; 3 Department of Nuclear Medicine, Hospitalsenheden Vest, Herning 7400, Denmark; 4 Department of Nuclear Medicine, Aarhus University Hospital, Aarhus 8000, Denmark

**Keywords:** ^99m^Tc-lactadherin, Lactadherin, ^99m^Tc-annexin V, Annexin V, Kinetics, Distribution, Clearance, Phosphatidylserine, Dosimetry

## Abstract

**Background:**

Phosphatidylserine (PS) is a phospholipid normally located in the inner leaflet of the cell membrane. PS is translocated from the inner to the outer leaflet of the plasma membrane during the early stages of apoptosis and in necrosis. In cell and animal studies, reversible PS externalisation to the outer membrane leaflet has been observed in viable cells. Hence, PS markers have been proposed as markers of both reversibly and irreversibly damaged cells. The purpose of this experimental study in pigs was to investigate the kinetics of the newly introduced PS marker technetium-99m-labelled lactadherin (^99m^Tc-lactadherin) in comparison with the well-known PS tracer ^99m^Tc-annexin V with special reference to the renal handling of the tracers. The effective dose for humans was estimated from the biodistribution in 24 mice.

**Methods:**

Nine anaesthetised pigs randomly allocated into two treatment groups were administered a single injection of either ^99m^Tc-lactadherin or ^99m^Tc-annexin V. Renal perfusion was assessed by simultaneous injection of ^51^Cr-EDTA. Throughout the examinations, planar, dynamic scintigraphy of the trunk was performed, urine was collected and arterial and renal vein blood was sampled. The effective dose was estimated using the adult male phantom from the RADAR website.

**Results:**

^99m^Tc-lactadherin was cleared four times faster from plasma than ^99m^Tc-annexin V, 57 ± 13 ml/min (mean ± SD) versus 14 ± 2 ml/min. ^99m^Tc-lactadherin had a predominant uptake in the liver, whereas ^99m^Tc-annexin V was primarily taken up by the kidneys. The estimated effective human dose after single injection of ^99m^Tc-lactadherin and ^99m^Tc-annexin V was 5.8 and 11 μSv/MBq, respectively.

**Conclusions:**

The high hepatic uptake of ^99m^Tc-lactadherin compromises the use of ^99m^Tc-lactadherin for imaging PS externalisation in the liver. Due to scatter from the liver, the use of *in vivo* visualisation of PS externalisation in the lower thorax and upper abdomen by ^99m^Tc-lactadherin is challenged, but not precluded. In contrast to ^99m^Tc-annexin, ^99m^Tc-lactadherin has a low renal uptake and may be the preferred tracer for imaging PS externalisation in the kidneys. The effective dose after injection of ^99m^Tc-lactadherin and ^99m^Tc-annexin was low. Recommendations regarding the clinical use of ^99m^Tc-lactadherin must await tracer kinetic studies in patients.

## Background

Phosphatidylserine (PS) is a phospholipid normally located in the inner leaflet of the cell membrane. PS is translocated from the inner to the outer leaflet of the plasma membrane during the early stages of apoptosis and in necrosis [1,2]. In cell and animal studies, reversible PS externalisation to the outer membrane leaflet has been observed in viable cells [3-5]. Hence, PS markers have been proposed as indicators of damaged cells, whether reversibly or irreversibly injured [6-8].

Fluorescent or radio isotope-labelled annexin V is frequently used to visualise the externalisation of PS on the cell surface [4,9,10]. Recently, the glycoprotein lactadherin (also known as MFG-E8) was introduced as a more sensitive PS marker [11,12], and the technetium-99m-labelled lactadherin (^99m^Tc-lactadherin) has been proven to readily visualise PS externalisation on cells [13].

The binding affinity of annexin V and lactadherin to cells with decreasing transmembrane potential is increased [14]. Since the membrane of apoptotic, hypoxic and ischemic cells depolarised, annexin V and lactadherin most likely bind to these cells with a higher affinity than to cells with preserved membrane potential.

The PS externalisation on dying cells is described as an ‘eat me’ signal to the macrophages that mediate engulfment [15]. Lactadherin facilitates this phagocytosis by acting as a bridge between exposed PS and the αvβ3 integrin on the macrophage surface [16]. It is well known that the inflammatory response to cell death is minimised when dying cells are effectively removed. A more direct anti-inflammatory effect of lactadherin has been demonstrated *ex vivo* by Voll et al. [17]. They showed that lactadherin increases the secession of anti-inflammatory cytokines from the macrophage. In experimental studies of inflamed and ischemic tissues, lactadherin is described as an organ-protecting component [18-20].

Given the promising future of lactadherin, both in diagnostic imaging and for anti-inflammatory treatment, knowledge of the kinetics of the compound is highly relevant.

The purpose of the present pharmacokinetic study in pigs was to compare the kinetics of ^99m^Tc-lactadherin with that of ^99m^Tc-annexin V with special reference to the renal handling. Further, the aim was to estimate an effective human dose by the use of data from an earlier biodistribution study in mice [21].

## Methods

### Animal preparation and experimental protocol

The study was approved by the Danish Inspectorate of Animal Experimentation and performed in accordance with their guidelines.

Nine female Danish Landrace/Yorkshire pigs, weighing 20 kg, were premedicated with midazolam 3 mg/kg (Dormicum®, Roche, Basel, Switzerland) and *s*-ketamine (*S*-Ketamin, Pfizer Aps, Ballerup, Denmark) subcutaneously. Subsequently, the pigs received a 375 mg bolus of pentobarbital (Mebumal®, DAK, Copenhagen, Denmark) in an auricular vein and were intubated and ventilated (Datex Ohmeda s/5 Avance ventilator, GE Healthcare, Little Chalfront, UK). During the entire data collection, anaesthesia was maintained by sevoflurane (Sevorane 100%, Abbotte Scandinavia AB, Solna, Sweden).

The arterial blood gases, pH, blood glucose and electrolytes were frequently monitored (ABL, Radiometer A/S, Brønshøj, Denmark), and ventilation was adjusted to keep the blood gases in physiological ranges. An intravenous saline infusion was maintained at a rate of approximately 200 ml · h^−1^. Temperature was registered with a rectal probe and was kept between 38°C and 39°C, which is the physiological range of temperature for pigs of this size. An appropriate environmental temperature was applied during transportation from the farm and during the study. If necessary, blankets and/or fans were used to maintain a steady, physiologic temperature.

A kidney vein catheter for venous blood samples was inserted through a sheath in the femoral vein. Since kidney vein blood has a higher oxygenation than mixed venous blood, the position of the catheter was assured by comparing the oxygen saturation in the kidney and jugular vein blood drawn simultaneously. Blood samples were drawn slowly from the kidney vein catheter to avoid aspiration of mixed venous blood from the inferior cava vein.

Arterial blood sampling and blood pressure measurement were performed through a sheath in the femoral artery. Heart rate and blood pressure were continuously monitored to ensure hemodynamic stability. A sheath was also inserted in the right internal jugular vein for tracer administration and saline infusion. The urinary bladder was catheterized and the urine collected.

The preparation time from the induction of anaesthesia to tracer injection was approximately 80 min.

### Tracer preparation and administration

The radio synthesis of ^99m^Tc-lactadherin and ^99m^Tc-annexin V has been described previously [13,21]. Five pigs received an injection dose (ID) of 19 to 38 MBq ^99m^Tc-lactadherin (^99m^Tc-hynic-lactadherin, Department of Nuclear Medicine, Denmark), and four pigs received 25 to 37 MBq ^99m^Tc-annexin V (^99m^Tc-hynic-recombinant human (rh)-annexin V, Reactionlab A/S, Lynge, Denmark) bolus in the jugular vein. The effective specific activity of ^99m^Tc-lactadherin and ^99m^Tc-annexin V at the time of injection was approximately 3.1 and 3.7 MBq/μg protein, respectively. The radiochemical purity was over 95% for both tracers. Simultaneously with PS tracer injection, all nine pigs received an intravenous dose of 4 MBq ^51^Cr-EDTA (Behring, Marburg, Germany) to determine the renal plasma flow (RPF).

### Dynamic scintigraphy

Dynamic imaging (64 × 64 matrix) of the trunk in the posterior projection was performed with a single-headed gamma camera (BrightView, Philips Medical, North Andover, MA, USA) equipped with a LEHR collimator. During the first 120 s post-injection (pi.), a frame rate of 1 per second was applied. From 2 to 60 min, the frame rate was 1 per minute, and from 60 min to the end of the study, 1 frame was recorded over 5 min (12 frames per hour).

The biodistribution of the PS tracers was evaluated by studying the tracer activity over time. Representative areas of the heart, lungs, liver, spleen and kidneys were drawn as regions of interest (ROI) on the scintigrams, and time/activity curves were generated. Activity was assessed as counts per minute per pixel and corrected for background radiation measured under the left kidney and for radioactive decay.

This method provides only a semi-quantitative description of tracer uptake in different organs over time, and the method is less suitable for quantitative comparison of tracer uptake between organs. Planar scintigraphy simplifies the three-dimensional tracer distribution to a two-dimensional image. Hence, organ activity assessed from planar images is proportional not only to tracer uptake, but also to the vertical thickness of the organs and the activity in overlying and underlying tissues.

### Blood and urine sampling

Blood samples (3 ml) were drawn from the renal vein and the femoral artery 2 min before tracer injection and at 2, 5, 10, 15, 20, 25, 30, 40, 50, 60, 75, 90, 105, 120, 135 and 150 min and then every 30th minute up to 240 min pi. Blood from two pigs, one injected with ^99m^Tc-annexin V and one with ^99m^Tc-lactadherin, were sampled up to only 150 min pi. Urine was collected simultaneously with blood sampling.

Radioisotope activity was counted in 1 ml plasma, 1 ml whole blood and 1 ml urine in a scintillation detector (Cobra II, Packard, Meriden, CT, USA) to a statistical accuracy of 1%. ^99m^Technetium activity was counted immediately after the experiment and ^51^Chromium activity 3 to 10 days later. Correction was made for background radioactive decay and cross-talk between channels.

### Calculations

The calculations were performed in three steps:

1. Whole blood clearance (Cl_Wb_), plasma clearance (Cl_P_), volume of distribution (*V*_d_), urinary clearance (Cl_u_), renal extraction (*E*_tracer_) and fraction of injected dose (ID) excreted in urine were calculated for all three tracers using the data collected from blood and urine samples.

2. RPF was calculated using the reference tracer ^51^Cr-EDTA and the equation

$${\mathrm{Cl}}_{\mathrm{r-EDTA}}=\mathrm{RPF}\times E_{\mathrm{EDTA}}$$

assuming that Cl_r-EDTA_ = urinary clearance (Cl_u-EDTA_).

3. Renal clearance (Cl_r-tracer_) of annexin and lactadherin was calculated from the equation

$${\mathrm{Cl}}_{\mathrm{r-tracer}}=\mathrm{RPF}\times E_{\mathrm{tracer}}$$

The fraction of the tracer retained in the kidneys was calculated as the relative difference in renal clearance (Cl_r-tracer_) and urinary clearance (Cl_u-tracer_) as a fraction of the renal clearance (Cl_r-tracer_).

#### Step 1

Whole blood and plasma clearances (Cl) were calculated as the ratio between the dose of the injected tracer (ID) and the total area under the time-activity curve [*P*(*t*)]:

$$\mathrm{Cl}=\mathrm{ID}/\int_{0}^{\infty }P\left(t\right)\mathrm{dt}$$

The area under the time-activity curve was calculated by fitting the time-activity data from the plasma and blood samples with three exponential functions using a peeling-off technique in a computer program (Sigma Plot 11, Systat Software, Chicago, IL, USA). Three exponential functions were used to get a satisfactory data fit. We did not use a compartment model.

Volume of distribution (*V*_d_) was assessed from the equation: $V_{d}=\bar{t}\times {\mathrm{Cl}}_{p}$, where $\bar{t}$ is the mean transit time and Cl_p_ is the plasma clearance of the tracer.

Urinary clearance (Cl_u_) was calculated according to the equation:

$${\mathrm{Cl}}_{u}=U\times V/\int_{0}^{240}P\left(t\right)\mathrm{dt},$$

where *U* denotes the concentration of the tracer in the urine and *V* the volume of urine collected. The product of *U* and *V* was divided by the area under the time-activity curve.

Renal extraction (*E*) was calculated from the relative difference in the plasma concentration of arterial blood (*P*_a_) and renal vein blood (*P*_v_):

$$E=\left(P_{a}-P_{v}\right)/P_{a}$$

The extraction was calculated for each sampling as well as an average value corrected to a mean time.

The fraction of ID excreted in urine is the amount of tracer found in urine divided by the ID.

#### Step 2

RPF was calculated as follows:

$$\begin{array}{l} {\mathrm{Cl}}_{\mathrm{r-EDTA}}=\mathrm{RPF}\times E_{\mathrm{EDTA}}\Rightarrow \\ \mathrm{RPF}={\mathrm{Cl}}_{\mathrm{r-EDTA}}/E_{\mathrm{EDTA}}, \end{array}$$

where Cl_u-EDTA_ was used as an estimate of Cl_r-EDTA_*.* EDTA is excreted solely by glomerular filtration, making Cl_u-EDTA_ a good estimate of Cl_r-EDTA_*.* Mean RPF was calculated for all nine pigs together and separately for pigs receiving either ^99m^Tc-annexin or ^99m^Tc-lactadherin.

#### Step 3

Renal clearance (Cl_r_) was calculated as follows:

$${\mathrm{Cl}}_{\mathrm{r-tracer}}=\mathrm{RPF}\times E_{\mathrm{tracer}}$$

Fraction retained in the kidneys was calculated using the equation:

$$\left({\mathrm{Cl}}_{r}–{\mathrm{Cl}}_{u}\right)/{\mathrm{Cl}}_{r}$$

### Dosimetry calculation on published data

The biodistribution of ^99m^Tc-lactadherin and ^99m^Tc-annexin V has been studied by our group in 24 mice sacrificed at 10, 60, or 180 min pi. [21]. From these data and by the use of the adult male phantom from the RADAR website (USA), an effective radiation dose to the human body after a single injection with the ^99m^Tc-lactadherin or the ^99m^Tc-annexin V compound was assessed.

## Results

### Results derived from blood and urine samples

**Figure 1:**
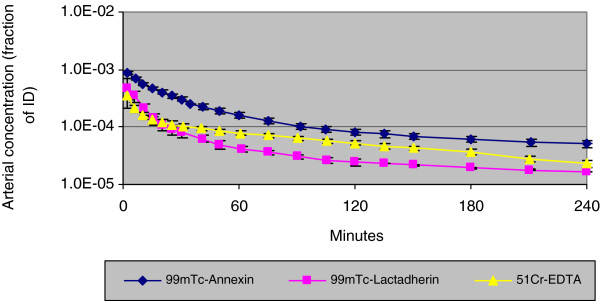
Mean arterial whole blood concentration (counts per minute/ml blood/ID) of the three tracers.

**Figure 2:**
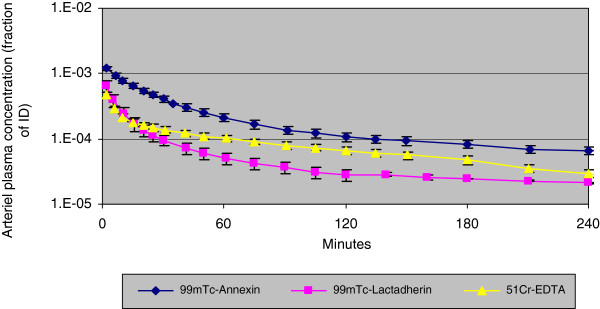
**Mean arterial plasma concentration** (**counts per minute**/**ml plasma**/**ID**) **of the three tracers.**

The whole blood and plasma disappearance curves of ^99m^Tc-lactadherin, ^99m^Tc-annexin V and ^51^Cr-EDTA are shown in Figures 1 and 2, respectively. During the first 15 min after injection, ^99m^Tc-lactadherin and ^51^Cr-EDTA concentration decreased almost in parallel, but from the 15th minute pi., the ^99m^Tc-lactadherin concentration decreased more rapidly than the EDTA concentration. During the entire study period, the decrease in the concentration of ^99m^Tc-annexin V was slower than that for the two other tracers. The main results from the calculations performed on the data from the blood and urine samples are listed in Table 1.

**Table 1:** Results derived from blood and urine samples

	**Lactadherin** (***n*** = **5**)	**Annexin** (***n*** = **4**)	**EDTA** (***n*** = **9**)
Plasma clearance (ml/min)	57 ± 13	14 ± 1.9	42 ± 6.8
Volume of distribution (L)	8.2 ± 3.6	3.3 ± 0.35	6.3 ± 1.2
Urinary clearance (ml/min)	2.4 ± 1.3	1.0 ± 0.37	28 ± 8.07
Renal extraction fraction	0.011 ± 0.004	0.037 ± 0.010	0.21 ± 0.05
Fraction of ID excreted in urine (120/180/240)	0.016/0.019/0.055	-/0.023/0.055	0.17/0.47/0.70
Urinary flow (ml/min)	1.7 ± 0.8	2.4 ± 1.4	2.0 ± 1.1
Renal plasma flow (ml/min)	152 ± 62	144 ± 22	148 ± 47
Renal clearance (ml/min)	1.6 ± 0.9	5.3 ± 1.5	30 ±6.0
Fraction retained in kidneys	−1.10 ± 1.3	0.80 ± 0.10	0.00

Data were calculated from blood and urine samples and presented as mean ± SD. Renal extractions were corrected to a mean time. Fraction excreted in urine at 120, 180 and 240 min after injection of tracer. Fractions retained in kidney were calculated as the amount of the injected dose cleared from the plasma by the kidneys. ID, injected dose; *n*, number.

**Figure 3:**
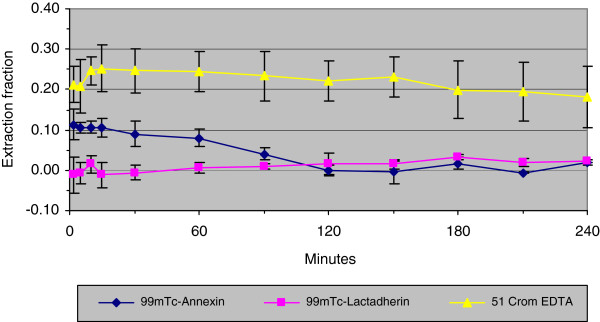
The mean renal plasma extraction fraction of the three tracers.

The renal extractions of the three tracers are shown in Figure 3. Mean renal extraction of ^99m^Tc-annexin V was initially 0.12 but close to zero 120 min pi. The mean renal extraction of ^99m^Tc-lactadherin was approximately zero throughout the experiment. EDTA's renal extraction was 0.25 initially, but the fraction gradually decreased to 0.18, 240 min pi.

### Scintigrams

**Figure 4:**
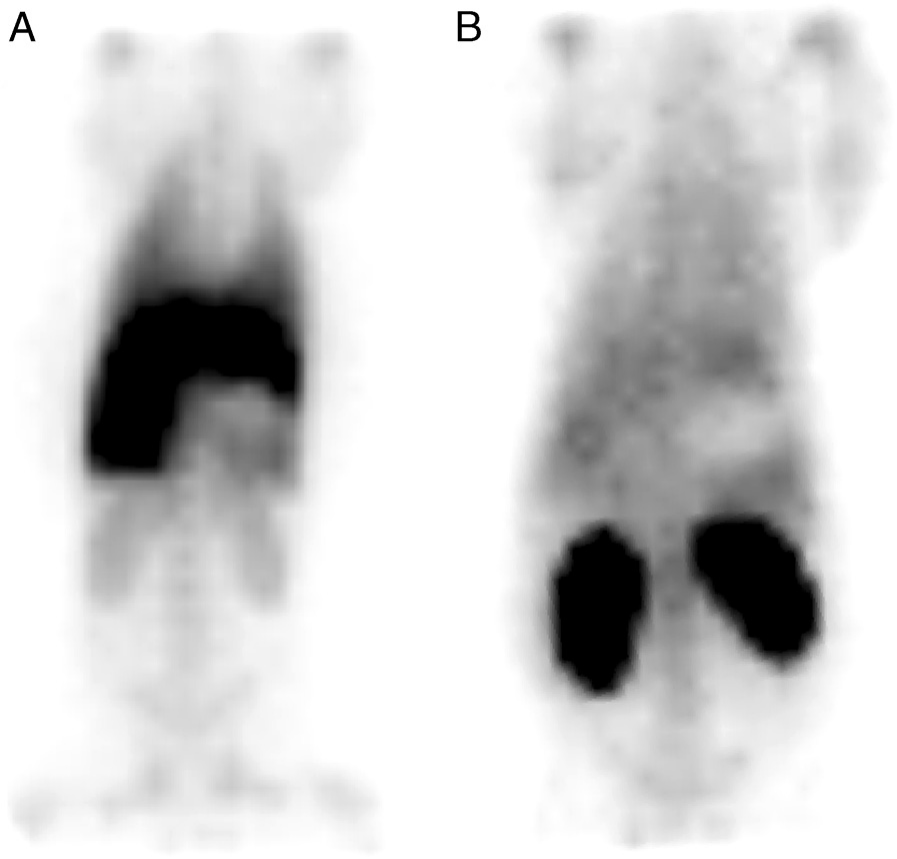
**Scintigrams of two representative pigs.** The scintigrams were obtained from 220 to 240 min after injection of (**A**) ^99m^Tc-lactadherin and (**B**) ^99m^Tc-annexin V.

The visual distribution of ^99m^Tc-lactadherin was quite different from the uptake pattern of ^99m^Tc-annexin V (Figure 4). The ^99m^Tc-annexin V was primarily taken up by the kidneys, whereas ^99m^Tc-lactadherin was predominately taken up in the liver.

**Figure 5:**
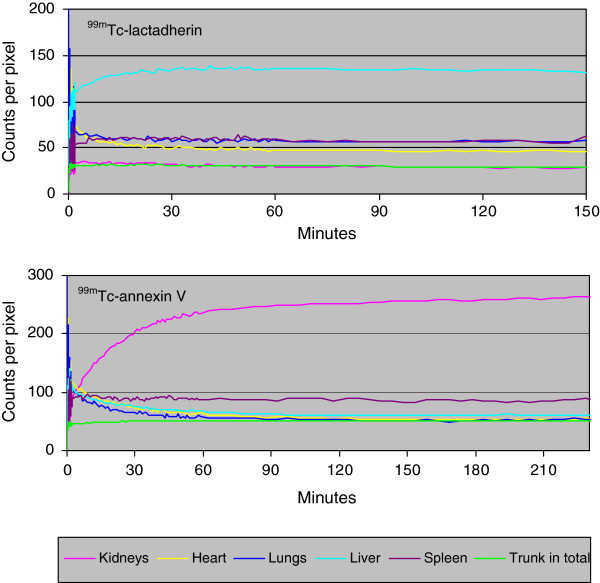
**Time**/**activity curves from ROIs for different organs from two representative pigs.** The curves illustrate the kinetics of ^99m^Tc-lactadherin and ^99m^Tc-annexin V.

The organ-specific time/activity curves of ^99m^Tc-lactadherin and ^99m^Tc-annexin V confirmed the difference between the two PS tracers' hepatic and renal uptake (Figure 5). Initially, ^99m^Tc-lactadherin accumulated rapidly in the liver, but from the 15th minute pi., the curve equalled out at a plateau approximately four times the average activity in the trunk, and 2.2 times the activity of the kidney. During the first hour pi., ^99m^Tc-annexin V accumulated in the kidneys before the kidney activity levelled out at a level four times higher than the liver and the average trunk activity.

### Effective dose estimated from biodistribution in mice

The effective dose for ^99m^Tc-lactadherin and for ^99m^Tc-annexin V was estimated from previously published data [21]. The biodistribution of ^99m^Tc-lactadherin in mice was found to be 64% in the liver, 4.7% in the kidneys, 12% in the blood and 19% in the remaining body. Effective residence times (worst case values) were estimated as 6, 6, 3.3 and 20 h, respectively. This biodistribution is assumed to be representative for humans, and by using dose factors from the adult male phantom from the RADAR website, the effective dose was estimated to be 5.8 μSv/MBq. The biodistribution of ^99m^Tc-annexin V was found to be 48% in the kidneys, 12% in the liver, 12% in the blood and 28% in the remaining body. Effective residence times (worst case values) were estimated as 6, 6, 4.4 and 18.6 h, respectively. The effective dose was calculated as 8.8 μSv/MBq.

## Discussion

The tracer kinetics of ^99m^Tc-annexin V has been studied in animal models as well as in humans. Clinical studies have shown the ability of ^99m^Tc-annexin V to visualise apoptosis, e.g. the complete or partial tumour response was associated with increased uptake of ^99m^Tc-annexin V [22-24].

^99m^Tc-lactadherin is a newer tracer with a higher affinity for the PS than ^99m^Tc-annexin V. Tracer kinetic studies have been performed only *in vitro* and in small animals [13,21]. We have made a comparative study of the kinetics of ^99m^Tc-lactadherin and ^99m^Tc-annexin V in pigs, an animal close to man both regarding the heart and the kidneys.

The present study showed important differences in the kinetics of ^99m^Tc-lactadherin and ^99m^Tc-annexin V. The renal extraction of ^99m^Tc-annexin V was only 3.7%, but 80% was retained in the kidneys and the kidneys were clearly delineated on the scintigrams. The renal clearance of ^99m^Tc-annexin V comprises nearly one third of the plasma clearance. Previous studies have also demonstrated that ^99m^Tc-annexin V was predominantly taken up by the kidney followed by liver and urinary bladder uptake [25]. The reason for the intense kidney accumulation remains unclear, but the uptake seems to be relatively nonspecific and does not alter upon apoptosis induction with cycloheximide in mice [26]. The same authors speculate that the reason might be an organ-specific endocytotic mechanism for annexin V that relies on a non-PS-dependent membrane association. The kidney uptake could have been free ^99m^Tc-pertechnetate. However, we did not see any activity in the thyroid, making this explanation less likely. The vague visualisation of the liver by ^99m^Tc-annexin V corresponded with previous findings [25], and it may be due only to the blood background.

A different pattern was observed for ^99m^Tc-lactadherin for which we observed a high hepatic uptake and only a minor uptake in the kidneys. The renal extraction of ^99m^Tc-lactadherin was as low as 1% and the renal clearance of ^99m^Tc-lactadherin responding to only 2.8% of the plasma clearance. The rapid and very dominant liver uptake corresponded well to the rapid plasma clearance of ^99m^Tc-lactadherin, the latest being more than four times faster than the plasma clearance of ^99m^Tc-annexin V. The distribution of the two PS tracers in the present study is in concordance with the results from the earlier study in mice [21].

The dominant liver uptake indicated that ^99m^Tc-lactadherin is either primarily metabolized in or slowly excreted by the liver as we did not observe a decline in the time/activity curve over the liver. Further, no bile ducts or bowl activity was visualised on the scintigrams, indicating that neither the isotope nor the tracer was excreted from the liver to a significant degree during the study periods.

Lactadherin is a glycoprotein identified in mammary glands, but also present in the brain, heart, lungs, spleen, intestines, liver, kidneys, reproductive organs and blood [27]. Notably, endogenously secretion might in many cases be ascribed to tissue-embedded macrophages [28]. This is in concordance with the suggested function of lactadherin, i.e. the glycoprotein operates as a bridging molecule between PS-exposing apoptotic/necrotic cells and integrin receptors on macrophages [16,29]. What hitherto is known about the nature of lactadherin prescribes that it most likely will be found as a membrane-associated component, especially the ones with high curvature [30], and suggests that under normal physiological conditions, blood lactadherin will be carried around on cell debris, membrane fragments or microvesicle-like structures. The liver has a well-developed ability to engulf apoptotic cells, involving the action of hepatocytes, Kupffer and endothelial cells [31]. This may explain the presently observed high and rapid liver uptake of ^99m^Tc-lactadherin.

One might argue that the high and rapid liver uptake of ^99m^Tc-lactadherin leaves no compound for targeting PS. However, in an ischemic and reperfused porcine model, we studied the PS externalisation in the myocardium after reperfusion using ^99m^Tc-lactadherin (RH Poulsen et al., unpublished work). A well-defined uptake of ^99m^Tc-lactadherin was found in the part of the myocardium exposed to ischemia and reperfusion, suggesting sufficient amount of ^99m^Tc-lactadherin for PS targeting despite the extensive hepatic uptake.

The effective dose for ^99m^Tc-lactadherin and for ^99m^Tc-annexin V was estimated from previously published data, assuming that the biodistribution in mice is representative for humans. The effective human dose after a single injection of ^99m^Tc-lactadherin was estimated to be 5.8 and 8.8 μSv/MBq for ^99m^Tc-annexin V which is in the clinically acceptable range and comparable to other routinely performed nuclear examinations. In comparison, in humans, the effective dose for ^99m^Tc-annexin V has been found to be 11 μSv/MBq [25].

^51^Cr-EDTA was used as a reference substance in order to calculate renal plasma flow to determine renal clearance of ^99m^Tc-lactadherin and ^99m^Tc-annexinV. We found a ^51^Cr-EDTA renal extraction of 21.6%, which is in concordance with earlier studies in pigs. During the experiment, the renal extraction of ^51^Cr-EDTA decreased slightly. Unlike humans, pigs have several kidney veins, which are both smaller and shorter than in humans. This makes blood sampling from the renal vein difficult. The concentration of ^51^Cr-EDTA is higher in the caval vein than in the renal veins, and contamination with caval blood would lead to an underestimation of the renal ^51^Cr-EDTA extraction. Contamination with blood from the inferior caval vein might explain the observed drop in EDTA excretion.

^51^Cr-EDTA is known to be excreted solely in the urine. The discrepancy between the plasma and urinary clearance of ^51^Cr-EDTA represents a bias that can be caused by the different methods used to estimate the two clearances.

The initial renal extraction fraction of ^99m^Tc-annexin V was more than 10% (Figure 3) but decreased to zero during the first 2 h of the experiment. Consequently, the contribution of the kidneys to the total plasma clearance decreased during the study period. On average, the renal extraction fraction of ^99m^Tc-annexin V was 3.7%.

The renal extraction fraction of ^99m^Tc-lactadherin was very close to zero throughout the study period. The amount of ^99m^Tc-lactadherin cleared from the blood on its way through the kidney was therefore very small. We found that the urinary clearance of ^99m^Tc-lactadherin exceeded the renal clearance. This has to be explained from the insignificant kidney excretion and the uncertainty of the measurement.

Overall, the results from the present tracer kinetic study in pigs were in accordance with the findings of recent studies in mice and humans [21,25]. Choosing a pig model was motivated by the knowledge of its genetic and physiological similarities to humans.

Further studies of the hepatic metabolism of ^99m^Tc-lactadherin are necessary to ensure a full understanding of the kinetic and biodistribution of the tracer. Moreover, it would be highly relevant to study ^99m^Tc-lactadherin's ability to visualise PS externalisation in apoptosis, ischemic or inflammatory experimental models. Finally, an adequate patient dosimetry study of ^99m^Tc-lactadherin is recommended before a potential clinical imaging with ^99m^Tc-lactadherin.

## Conclusions

The high hepatic uptake of ^99m^Tc-lactadherin may compromise the use of ^99m^Tc-lactadherin for visualisation of PS externalisation in the liver and in organs close to the liver. In contrast to ^99m^Tc-annexin, ^99m^Tc-lactadherin has a low renal uptake and may be the preferred tracer for imaging PS externalisation in the kidneys. The biodistribution of ^99m^Tc-annexin V favours this tracer as the preferred marker for imaging PS externalisation in the upper abdomen and the lower thorax.

The estimated effective human dose after a single injection of ^99m^Tc-lactadherin is in the clinically acceptable range and comparable to other routinely performed nuclear examinations. However, recommendation regarding the clinical use of ^99m^Tc-lactadherin must await tracer kinetic studies and dosimetry calculations in patients.

## Competing interests

The authors declare that they have no competing interests.

## Authors’ contributions

RHP carried out the experimental studies and participated in the analysis of data. JTG participated in the design of the study. JAE participated in the design of the study, carried out the experimental studies and analysed the data. CF calculated the estimation of the dosimetry. LF carried out the tracer preparation. CWH participated in the design of the study. MR participated both in designing the study and in analysing the data. RHP, JTR, CWH and RM drafted the manuscript. All authors read and approved the final manuscript.
